# Comparison of muscle synergies in walking and pedaling: the influence of rotation direction and speed

**DOI:** 10.3389/fnins.2024.1485066

**Published:** 2024-12-04

**Authors:** Junko Tsuchiya, Kimito Momose, Hiroki Saito, Koji Watanabe, Tomofumi Yamaguchi

**Affiliations:** ^1^Major of Physical Therapy, Department of Rehabilitation, School of Health Sciences, Tokyo University of Technology, Tokyo, Japan; ^2^Department of Health Sciences, Graduate School of Medicine, Shinshu University, Nagano, Japan; ^3^Department of Physical Therapy, School of Health Science, Shinshu University, Nagano, Japan; ^4^Centre for Human Movement at Tokyo University of Technology, Tokyo, Japan; ^5^Department of Rehabilitation Medicine, Juntendo University Graduate School of Medicine, Tokyo, Japan; ^6^Department of Physical Therapy, Human Health Sciences, Graduate School of Medicine, Kyoto University, Kyoto, Japan; ^7^Department of Physical Therapy, Faculty of Health Science, Juntendo University, Tokyo, Japan

**Keywords:** locomotor modules, gait, cycling, electromyography (EMG), motor control, rehabilitation, central nervous system (CNS)

## Abstract

**Background:**

Understanding the muscle synergies shared between pedaling and walking is crucial for elucidating the mechanisms of human motor control and establishing highly individualized rehabilitation strategies. This study investigated how pedaling direction and speed influence the recruitment of walking-like muscle synergies.

**Methods:**

Twelve healthy male participants pedaled at three speeds (60 RPM, 30 RPM, and 80 RPM) in two rotational directions (forward and backward). Additionally, they completed walking tasks at three different speeds (slow, comfortable, and fast). Surface electromyography (EMG) was recorded on 10 lower limb muscles during movement, and muscle synergies were extracted from each condition using non-negative matrix factorization. The similarities between the muscle synergies during walking and each pedaling condition were examined using cosine similarity.

**Results:**

The results confirmed that the composition of muscle synergies during pedaling varied depending on the rotational direction and speed. Furthermore, one to three muscle synergies, similar to those observed during walking, were recruited in each pedaling condition, with specific synergies dependent on direction and speed. For instance, synergy involving the quadriceps and hip extensors was predominantly observed during pedaling at 30 RPM, regardless of the direction of rotation. Meanwhile, synergy involving the hamstrings was more pronounced during forward pedaling at 60 RPM and backward pedaling at 80 RPM.

**Conclusion:**

These findings suggest that walking-like muscle synergies can be selectively recruited during pedaling, depending on the rotational direction and speed.

## 1 Introduction

Human motor control is a highly complex behavior, with the central nervous system (CNS) controlling vast degrees of freedom of the musculoskeletal system (Bernstein, 1967). Muscle synergies are functional units consisting of groups of muscles that work together in a coordinated manner to control specific motor tasks. To address the excessive redundancy in motor control, the CNS has been suggested to achieve complex movements by flexibly combining a small number of fundamental muscle synergies (Tresch and Jarc, 2009; Bizzi and Cheung, 2013; Ting et al., 2015).

Walking and cycling involve rhythmic movements of the lower limbs and exhibit similar muscle activity patterns (Raasch and Zajac, 1999) and neural modulation (Zehr et al., 2007). This similarity suggests a shared neural control mechanism and comparable muscle synergies between walking and pedaling have also been noted (Hug et al., 2010; De Marchis et al., 2013; Barroso et al., 2013, 2014). The activity of multiple muscle groups during walking can be explained by four or five muscle synergies (Ivanenko et al., 2004; Ivanenko et al., 2005; Cappellini et al., 2006; Neptune et al., 2009; Clark et al., 2010). Additionally, muscle activity during pedaling is often explained by three or four muscle synergies (Hug et al., 2010; Hug et al., 2011; De Marchis et al., 2013; Barroso et al., 2013, 2014; Ambrosini et al., 2016). However, a study investigating muscle synergies during pedaling at various cadences showed that although the number of required synergies remains consistent across different cadences, the composition of the recruited muscle synergies varies with speed (Barroso et al., 2014).

Barroso et al. (2014) compared muscle synergies between walking and pedaling at four different speeds and reported that similar muscle synergies were observed between these two motor tasks. However, they noted that a muscle synergy in which the soleus muscle contributed independently was only observed during low-speed pedaling (Barroso et al., 2014). Alternatively, during walking, a muscle synergy involving the ankle plantar flexors, which contribute to forward propulsion during the late stance phase, has been observed across all speeds from low to high (Ivanenko et al., 2004; Clark et al., 2010; Yokoyama et al., 2016). Barroso’s study investigated muscle synergies in pedaling at four speeds, comparing them with walking synergies at matched speeds. Their findings suggest that muscle synergies essential for walking may be observed only during pedaling at specific speeds. Therefore, it is important to investigate the similarity of muscle synergies during walking and during pedaling at different speeds.

Furthermore, a simulation study examining the contribution of functional muscle groups in different pedaling directions (forward and backward) reported that smooth backward pedaling was achieved by splitting the pairs of the rectus femoris (RF)/tibialis anterior (TA) and hamstrings (HAM)/ankle plantar flexors (TS) observed in forward pedaling into two distinct pairs: RF-HAM and TA-TS pair (Raasch and Zajac, 1999). However, given the findings of previous studies that the composition of muscle synergies during pedaling varies with movement direction and speed (Barroso et al., 2014), it is possible that the composition of walking-like muscle synergies recruited during pedaling may also differ depending on these factors.

Pedaling exercises are widely used in the rehabilitation of patients with stroke as an effective method for regaining walking function, including improvements in walking speed, distance, and asymmetry (Barbosa et al., 2015). Clarifying the details of the shared muscle synergies between pedaling and walking is essential for assessing motor function impairments and establishing highly individualized rehabilitation strategies. This study aimed to investigate the similarities between muscle synergies obtained from six pedaling conditions, combining forward and backward pedaling at three different speeds and walking muscle synergies, to elucidate the composition of walking-like muscle synergies recruited during pedaling based on direction and speed.

## 2 Materials and methods

### 2.1 Subjects

Twelve healthy male volunteers (age, 25 ± 2; height, 1.71 ± 0.06 m; weight, 68.0 ± 7.5 kg) participated in this study. These individuals met the inclusion criteria of no history of CNS disorders or orthopedic conditions that would impair walking or pedaling movements. Individuals with training experience as cyclists were excluded.

They were informed about all procedures and the potential discomfort associated with the experimental procedures before providing written consent to participate. The study protocol was approved by the Institutional Review Board of Shinshu University, Nagano, Japan (Approval No. 4473) and adhered to the standards of the latest revision of the Declaration of Helsinki.

### 2.2 Experimental procedure

The participants completed three sessions. In the first session, the participants walked on flat ground at three speeds: comfortable, slow, and fast. Next, forward pedaling was performed on a recumbent ergometer (StrengthErgo240; Mitsubishi Electric Co., Tokyo, Japan) at three speeds: comfortable, slow, and fast. Finally, they pedaled backward on the same ergometer at three speeds.

### 2.3 Gait

The participants walked on flat ground at three different speeds, and each speed was recorded once after the preparer practiced. Measurements were conducted using a 16 m walking path, which included three meters before and after the acceleration and deceleration sections. Pressure signals during walking were recorded from a foot switch attached to the right heel during a 10 m section where the walking speed was constant. The timing of the heel strike was used to identify one walking cycle and cadence.

### 2.4 Pedaling

Pedaling was performed using a recumbent ergometer with an adjustable seat height of 51 cm and a crank length of 18 cm. The backrest angle was set to 10°, and the distance from the seat to the crank axis and the height of the pedal axis were adjusted to ensure the knee extension angle was −10° when the knee was maximally extended during pedaling.

**Figure 1:**
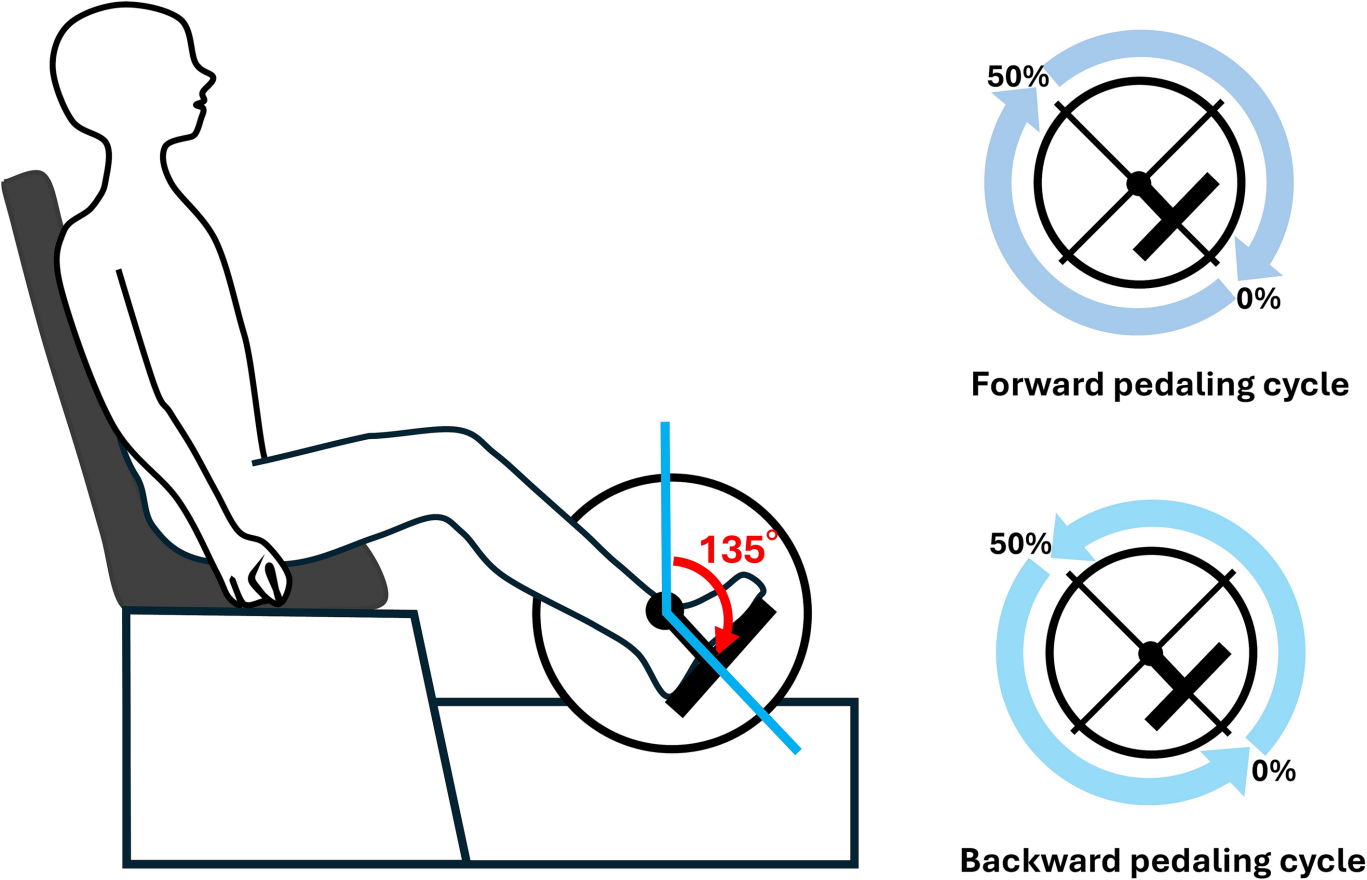
Experimental setup. As illustrated in the figure, the starting position of the pedaling cycle was defined as the right crank angle at 135°. Both forward and backward pedaling were recorded. One segment was defined as the period from the initial 135° position until it reached 135° again, and this segment was represented as 100% of the pedaling cycle.

The participants pedaled during all tasks using an isometric contraction mode of 10 Nm. Pedaling speeds (expressed in revolutions per minute, RPM) were selected at 60 RPM, 30 RPM, and 80 RPM, with the aim of capturing a wide range of differences in muscle synergies induced by pedaling speed. The rationale for selecting these specific speeds was based on previous studies involving stroke patients that employed slower pedaling speeds, ranging from 20 to 50 RPM (Ambrosini et al., 2016), while research on trained cyclists utilized higher speeds, ranging from 60 to 140 RPM (Wakeling and Horn, 2009). Based on these methodologies and considering the ability of our participants to maintain a steady pedaling cadence without undue strain, we selected 30 RPM as a slower speed and 80 RPM as a faster speed. Additionally, the comfortable cadence was set at 60 RPM, per previously established methods (De Marchis et al., 2013). The order of the speeds in each session was randomized. A 30 s trial was conducted for each speed of forward and backward pedaling. Real-time measurement of the crank angle during pedaling was made possible by recording voltage changes from the left crank. Since all participants in this study were right-leg dominant, the right-side profiles were shifted by 180°. The pedaling cycle in this study was defined as starting when the right knee transitioned from the extension phase to the flexion phase (the right crank angle at 135°) and ending after the completion of one full revolution (Figure 1).

### 2.5 Electromyogram

Surface electromyography (EMG) was recorded from the following 10 muscles of the dominant lower limb: tibialis anterior (TA), soleus (SOL), lateral gastrocnemius (LG), rectus femoris (RF), vastus medialis (VM), vastus lateralis (VL), medial hamstrings (MH), lateral hamstrings (LH), gluteus medius (Gmed), and gluteus maximus (Gmax). Based on previous studies (Barroso et al., 2014) investigating muscle synergies during cycling, which demonstrated consistent EMG patterns from the dominant leg, this study also focused on measuring muscle activity from the dominant leg. Participants underwent gait and pedaling tasks focusing on their dominant side, and each participant’s dominant leg was established using the Footedness Questionnaire (Chapman et al., 1987). Electrode placement followed the SENIAM (surface electromyography for the noninvasive assessment of muscles) guidelines (Hermens et al., 2000). EMG activity was recorded during a stable performance of each task using a wireless EMG system (Trigno Wireless System; DELSYS, Boston, MA, United States). The EMG signals were bandpass-filtered (20–450 Hz), amplified (with a 300-gain preamplifier), and sampled at 2000 Hz. Data analysis was conducted offline using MATLAB R2022b (MathWorks, Natick, MA, United States) and IBM SPSS Statistics 25 software (IBM).

### 2.6 EMG processing

Before commencing EMG processing, a meticulous visual inspection of the EMG recordings from all muscles was conducted. A continuous series of six strides/pedaling cycles devoid of noise artifacts was carefully selected for analysis in each trial. Selected EMG signals underwent full-wave rectification and were smoothed using a Butterworth zero-phase low-pass filter with a cutoff frequency of 5 Hz (Clark et al., 2010; Hug et al., 2010; Barroso et al., 2014).

The smoothed EMG data were normalized to the average of the peaks from each muscle’s six strides/pedaling cycles to facilitate comparisons across subjects, motor tasks, and speeds. Additionally, the EMG signals were resampled at intervals of 200 points for each stride/pedaling cycle (Barroso et al., 2014). Furthermore, we subtracted the minimum value for each cycle to ensure a zero value for all cycles (Barroso et al., 2014). For each subject, motor task, and speed, the normalized EMG signals were combined into an *m* × *t* matrix, where *m* represents the number of muscles (10 in this case), and *t* indicates the time base (*t* = number of strides (6) × 200 timepoints) (Barroso et al., 2014).

### 2.7 Muscle synergy analysis

To extract the motor modules, non-negative matrix factorization (NMF) was performed on the EMG matrices (EMG_0_) obtained from each trial, consisting of six continuous cycles for each participant (Clark et al., 2010; Hug et al., 2010; Barroso et al., 2014). NMF is a linear decomposition technique that decomposes a given data matrix into two non-negative matrices, as represented by the following equation (Lee and Seung, 1999; Tresch et al., 2006):


M=W⋅C+e

where *M* represents an *m* × *t* matrix (i.e., 10 muscles × 1,200 time points, comprising six cycles × 200-time points), *W* is an *m* × *n* matrix representing the weighting components (where *n* is the number of modules), *C* is an *n* × *t* matrix representing the temporal pattern components, and *e* is the residual matrix. When the matrices *W* and *C* are multiplied, an *m* × *t* matrix is generated that attempts to reconstruct the EMG for all consecutive cycles.

At each iteration, the algorithm updates *W* and *C* to minimize the Frobenius norm representing the residual between the reconstructed EMG (EMG_r_) and original EMG matrix (EMG_0_) (Lee and Seung, 1999). NMF was applied to all possible *n* values, ranging from 1 to 10, for module extraction. Muscle synergy vectors (columns of matrix *W*) were normalized by the maximum value of each column to enable comparisons among the subjects, speeds, and motor tasks (Hug et al., 2010; Barroso et al., 2014). In addition, each row of matrix *C* was normalized to its peak for all cycles.

As the algorithm iteratively updates based on random initial estimates of *W* and *C*, it converges to a locally optimal matrix factorization. To avoid the local minima, the algorithm was repeated 100 times for each participant. The variance accounted for (VAF) was calculated at each iteration, and only the iteration with the maximum VAF was retained. VAF is defined as follows:


$$VAF=1-\frac{\sum_{i=1}^{m}\sum_{j=1}^{l}{\left(EMG0\left(i,j\right)-\mathrm{EMGr}\left(i,j\right)\right)}^{2}}{\sum_{i=1}^{m}\sum_{j=1}^{l}{\left(EMG0\left(i,j\right)\right)}^{2}}$$

We defined the optimal number of modules, *n*, as meeting the following criteria: first, *n* was selected as the smallest number of modules, explaining more than 90% of the VAF (Torres-Oviedo et al., 2006). Second, *n* was the smallest number, and adding another module did not increase the VAF by more than 5% (Frère and Hug, 2012).

Daily life requires walking at a wide range of speeds, from slow to fast, and stroke patients undergoing rehabilitation need to regain their walking ability across this range of speeds. Therefore, in this study, we compared walking across a broad range of speeds with pedaling at various speeds, considering its potential application for rehabilitation.

To this end, we concatenated the EMG matrices obtained from each subject walking at three speeds (comfortable, slow, and fast) along the time points in the direction. Subsequently, NMF was performed on the concatenated EMG matrix of walking at all speeds (i.e., the matrix consisted of 10 muscles × 3 speed conditions × 1,200 time points) to extract the synergies across all walking speeds (Yokoyama et al., 2016; Saito et al., 2021).

### 2.8 Clustering the muscle synergy across participants

To elucidate the characteristics of muscle synergy vectors among the different conditions, hierarchical clustering analysis (Ward’s method, Euclidean distance) was conducted on the weighting components of the muscle synergies for all subjects in each condition (Yokoyama et al., 2016; Saito et al., 2021). Clustering was performed for each of the seven conditions: three velocities for forward pedaling, three velocities for backward pedaling, and whole-speed walking. The optimal number of clusters was determined using a gap statistic (Tibshirani et al., 2001). Subsequently, the muscle synergy vectors within the clusters were averaged across the subjects. Synergies possessed by more than half of the subjects were defined as representative synergies for each condition (Funato et al., 2022) and were adopted for further examination of similarities with walking synergies. The similarity between walking and representative synergies for each pedaling condition was assessed using cosine similarity, and synergies were considered similar when the cosine similarity was more significant than 0.85.

### 2.9 Statistical analyses

All statistical analyses were performed using IBM SPSS Statistics 25 software (IBM). The Wilcoxon signed-rank test, which is appropriate for paired data, was used to compare the differences in cadence across the walking speed conditions (comfortable, slow, fast). All statistical significance levels were set at *p* ≤ 0.05.

## 3 Results

### 3.1 Walking cadences

The cadences (mean ± standard deviation) at each walking speed (comfortable, slow, fast) were 59 ± 2 strides/min, 51 ± 4 strides/min, and 69 ± 4 strides/min, respectively. Significant differences among the cadence conditions were observed in the comfortable vs. slow, slow vs. fast, and comfortable vs. fast conditions (*p* < 0.001, Wilcoxon signed-rank test).

### 3.2 Muscle synergies extracted from whole-speed walking EMG matrices

Table 1 presents the VAF values of the 12 subjects for each condition. The median number of muscle synergies required to meet the criteria for whole-speed walking was 4.5, and the mean VAF for the optimal number of synergies across all subjects was 94.2 ± 2.2%.

**Table 1:** The number of muscle synergies and VAF in each condition.

	Whole-speed walking	Forward pedaling			Backward pedaling		
		60 RPM	30 RPM	80 RPM	60 RPM	30 RPM	80 RPM
Median (min, max)	4.5 (3, 5)	4 (4, 5)	4 (3, 5)	4 (4, 5)	4 (2, 5)	4 (2, 5)	4 (3, 5)
VAF	94.2 ± 2.2	97.9 ± 1.1	96.9 ± 1.1	98.5 ± 1.0	96.9 ± 2.0	96.9 ± 2.1	97.3 ± 1.2

This table shows the number of muscle synergies required to explain the electromyogram (EMG) patterns during whole-speed walking and each pedaling condition, along with the corresponding variance accounted for (VAF) values. The number of muscle synergies is presented as the median (maximum, minimum) across all subjects, and the VAF is presented as the mean ± standard deviation (SD).

**Figure 2:**
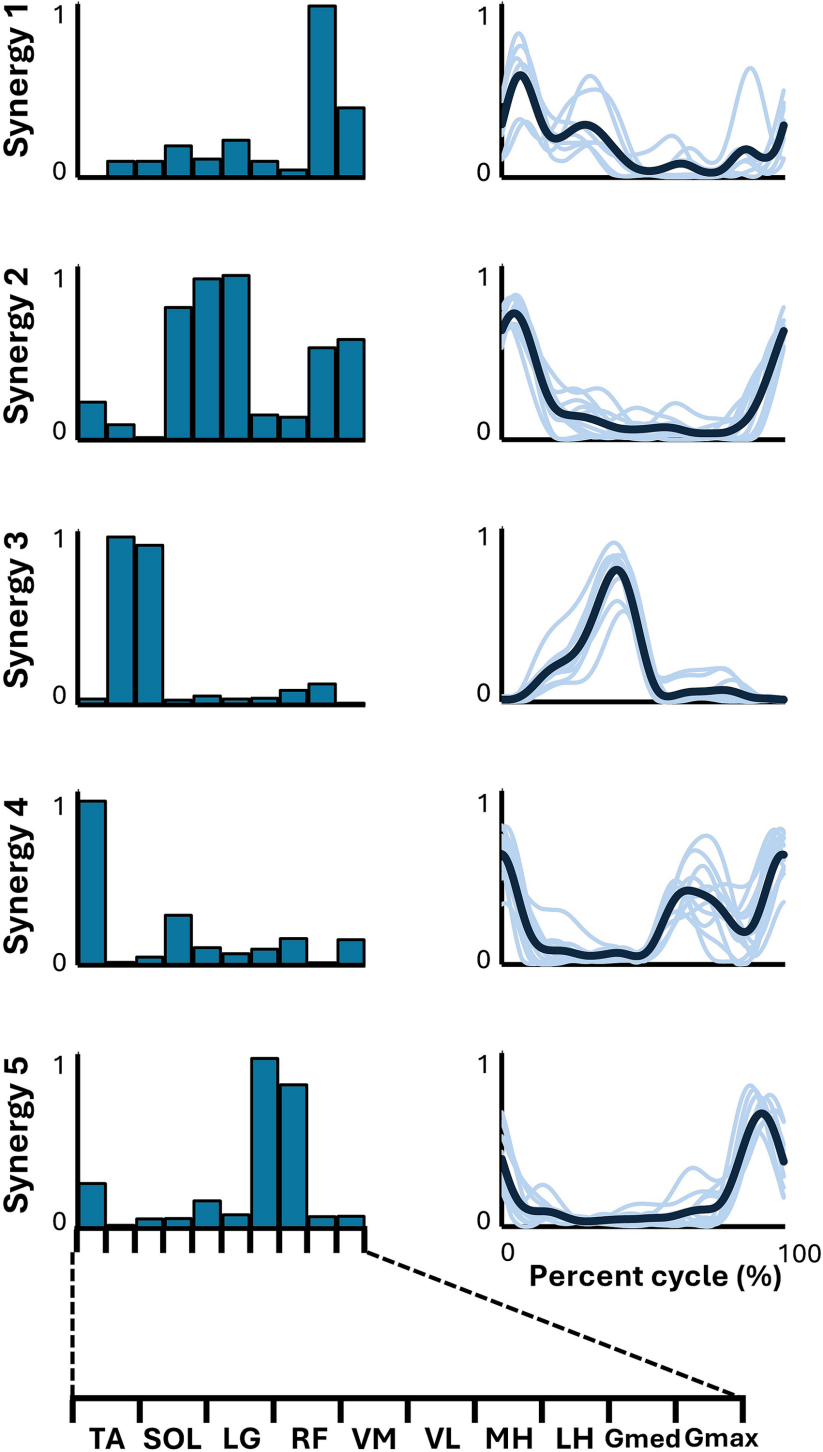
Synergies from whole-speed walking. The representative muscle synergies (bar graphs) and corresponding temporal patterns (bold lines) during whole-speed walking are shown. The representative muscle synergies for each condition are arranged based on the timing of the peak in the temporal patterns. The thin lines in the temporal patterns indicate the mean values over six cycles for each subject.

Figure 2 illustrates the representative synergies (mean muscle synergy vectors within each cluster) and corresponding average temporal pattern components during whole-speed walking. Table 2 lists the muscles that primarily contribute to each representative synergy based on visual inspection and the number of subjects within each cluster.

**Table 2:** Characteristics of muscle synergies and the number of subjects within the cluster synergies.

		Number of participants within clusters		
Whole-speed walking			Forward pedaling	Backward pedaling
Synergy	Major muscles	Whole-speed walking	60 RPM/30 RPM/80 RPM	60 RPM/30 RPM/80 RPM
Synergy1	Gmed	7	-/-/-	-/3/-
Synergy2	Quad, Gmed, Gmax	11	-/12/-	-/12/-
Synergy3	SOL, LG	10	5/8/5	5/7/7
Synergy4	TA	12	11/10/12	6/6/9
Synergy5	MH, LH	12	6/-/-	5/4/11

This table shows the muscles with the greatest contribution to each synergy during whole-speed walking, the number of subjects in which each synergy was observed, and the number of subjects in which walking-like muscle synergies were observed under each pedaling condition.

The lower limb EMG activities during walking, encompassing speeds from slow to fast, were adequately explained by five muscle synergies for all participants. Additionally, all five muscle synergies were shared by more than half of the participants. Therefore, for the comparison of similarities with each pedaling condition, we adopted the five muscle synergies as representative muscle synergies for whole-speed walking.

### 3.3 Muscle synergies in various pedaling conditions

**Figure 3:**
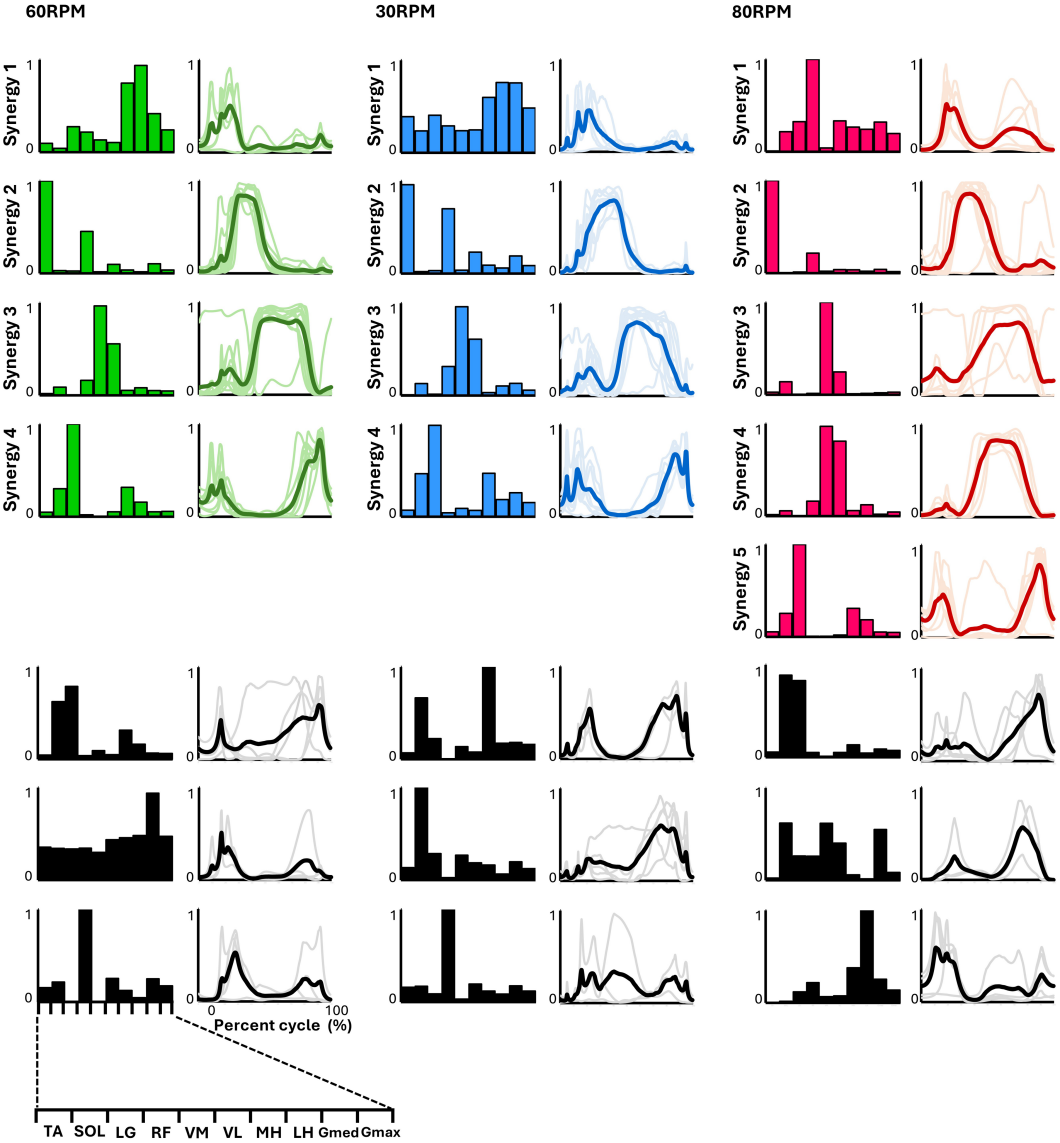
Synergies from forward pedaling. The representative muscle synergies (bar graphs) and corresponding temporal patterns (bold lines) during forward pedaling are shown. The representative muscle synergies for each condition are arranged based on the timing of the peak in the temporal patterns. The thin lines in the temporal patterns indicate the mean values over six cycles for each subject. The black graphs shown represent subject-dependent muscle synergies, which were observed in fewer than half of the subjects.

In all six pedaling conditions, the median number of synergies required to meet the criterion was consistently four (Table 1). Figure 3 shows the representative synergies and corresponding average temporal pattern components for each speed during forward pedaling. The muscle synergy vectors from all subjects during each forward pedaling condition were clustered into seven groups at 60 RPM and 30 RPM and into eight groups at 80 RPM. Among these, four representative synergies were identified at 60 and 30 RPM and five at 80 RPM.

**Figure 4:**
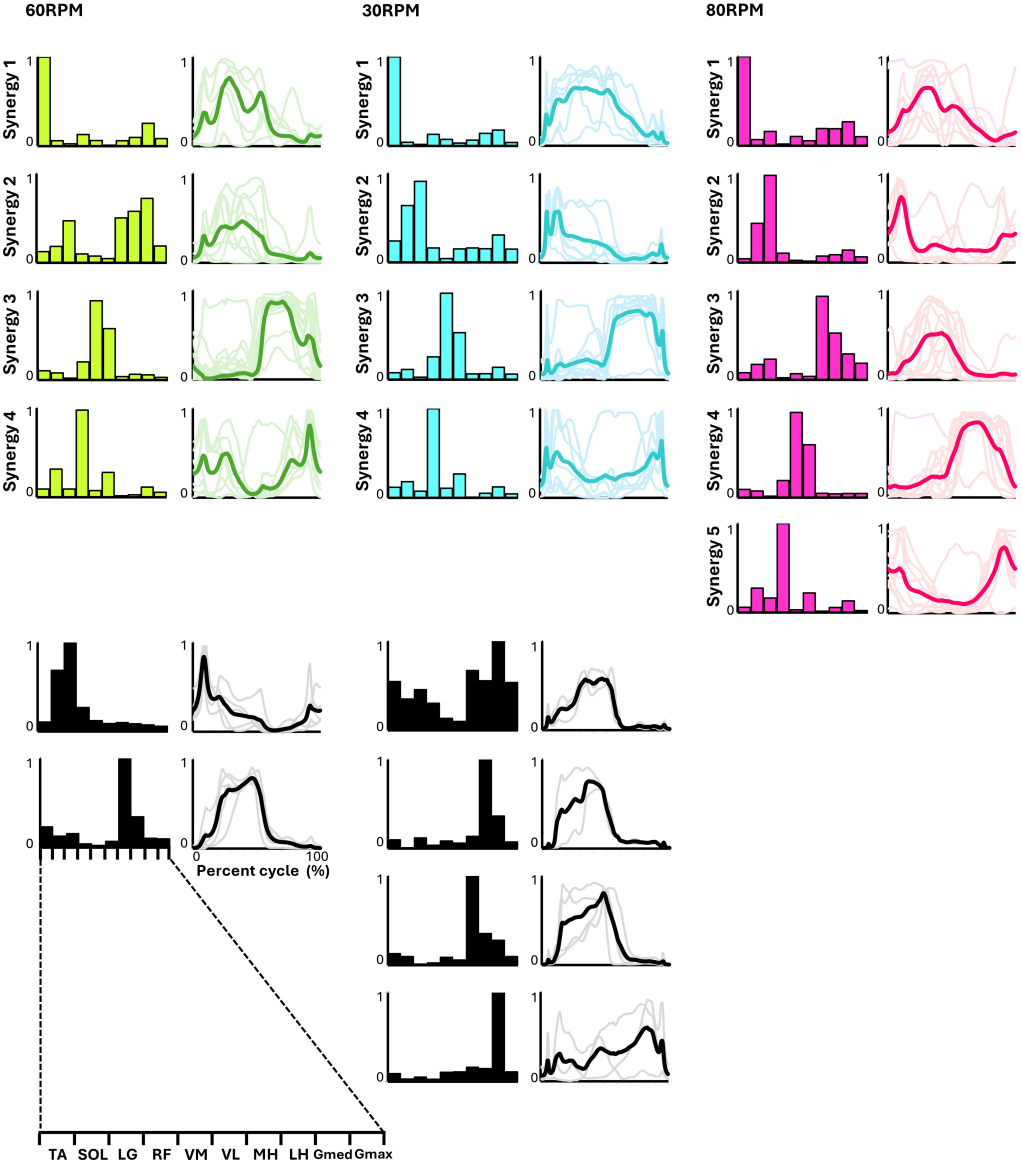
Synergies from backward pedaling. The representative muscle synergies (bar graphs) and corresponding temporal patterns (bold lines) during backward pedaling are shown. The representative muscle synergies for each condition are arranged based on the timing of the peak in the temporal patterns. The thin lines in the temporal patterns indicate the mean values over six cycles for each subject. The black graphs shown represent subject-dependent muscle synergies, which were observed in fewer than half of the subjects.

Figure 4 presents the representative synergies and corresponding average temporal pattern components for each speed during backward pedaling. Muscle synergy vectors from all subjects during each backward pedaling condition were clustered into six, eight, and five groups at 60, 30, and 80 RPM, respectively. Among these, four representative synergies were observed at 60 and 30 RPM and five at 80 RPM.

Pedaling conditions, except for backward pedaling at 80 RPM, exhibited subject-specific muscle synergies. Notably, numerous subject-specific muscle synergies were observed at backward pedaling at 30 RPM. This suggests that the muscle synergies recruited during pedaling are less robust and show greater variability among individuals compared to those during walking.

### 3.4 The similarity between walking muscle synergies and pedaling muscle synergies

**Figure 5:**
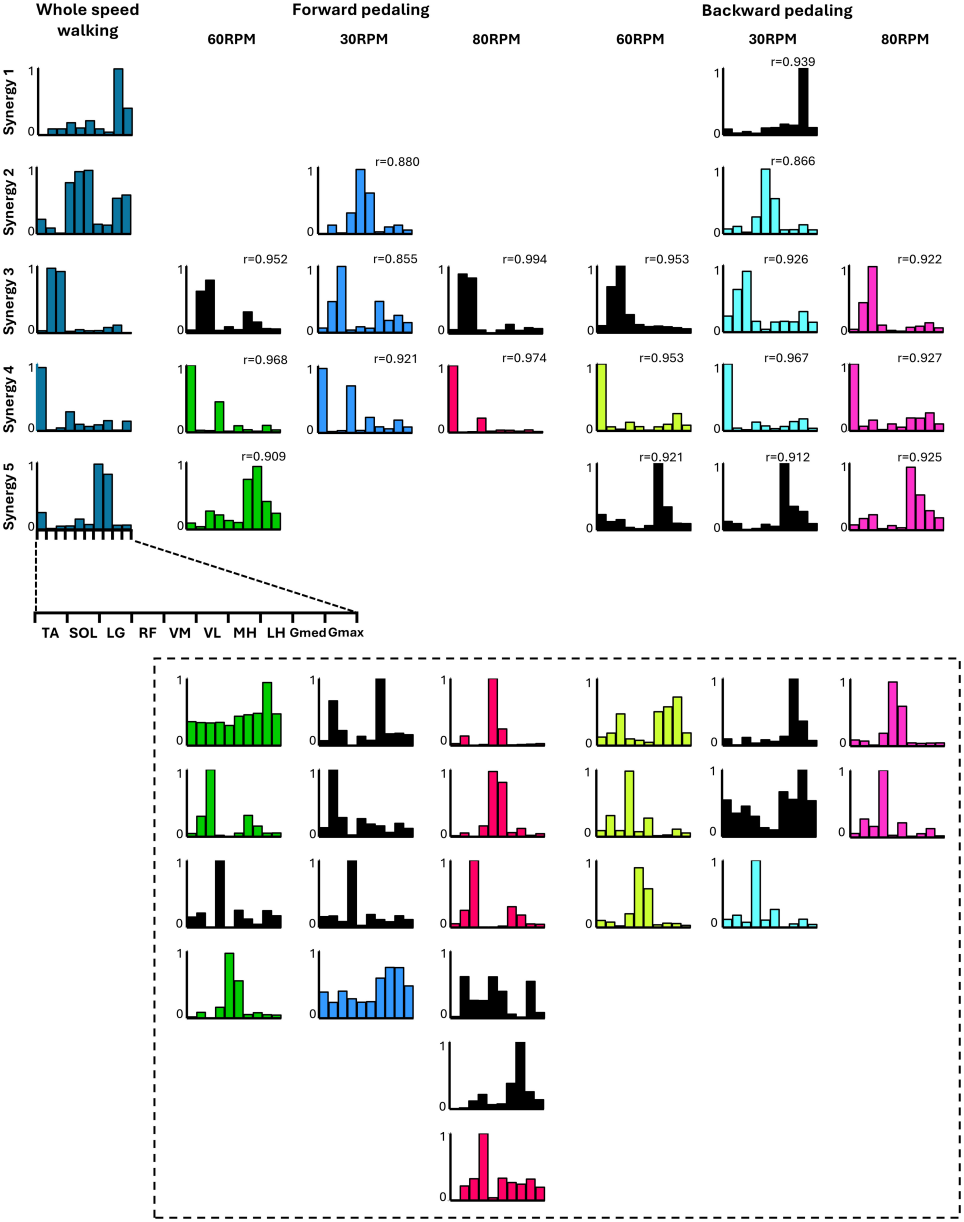
The similarity between the five muscle synergies from the whole-speed walking dataset of all subjects and the weighting components of each pedaling condition. For each condition, while the required number of essential modules remained the same, the composition of the weighting component of representative muscle synergies differed. Each bar graph represents the centroid of each cluster of weighting components obtained from each condition. Within each column, synergies obtained from each condition are arranged with bars of the same color. The black graph represents synergies specific to individual subjects (i.e., observed in less than half of the samples). The leftmost column represents the representative synergies (synergy 1 to synergy 5) of whole-speed walking. Synergy 1 consisted of the activation of the Gmed and Gmax during the early to mid-stance phase. Synergy 2 was characterized by the activity of the quadriceps group, Gmed, and Gmax during the early stance phase. Synergy 3 involved the activation of SOL and LG during the late stance phase. Synergy 4 was composed of TA and RF activities during both the early and late swing phases. Finally, synergy 5 included the activity of the hamstrings and TA from the late swing to early stance phase. The *r* value in the graph represents the cosine similarity between each synergy and the corresponding representative muscle synergy from whole-speed walking. Similar synergies obtained from each pedaling condition (*r* ≥ 0.85) are displayed in correspondence with the rows of synergies 1 to 5. Synergies enclosed by dashed lines below are those with low similarity to walking synergies, indicating task-specific synergies.

Figure 5 summarizes the representative synergies of whole-speed walking as a reference and the representative and subject-dependent muscle synergies for each pedaling condition (forward and backward pedaling), sorted by cosine similarity. Table 2 shows the representative muscle synergies of walking, the primary contributing muscles, and the number of participants contributing to the pedaling muscle synergies sorted for each walking synergy. In each of the six pedaling conditions, one to three sets of muscle synergies similar to those of walking were identified, with the composition within each set varying depending on the combination of rotation direction and speed. Muscle synergies similar to walking synergy 2 were observed only at a rotational speed of 30 RPM in both forward and backward pedaling. In contrast, muscle synergies similar to synergy 5 were observed only during forward pedaling at 60 RPM and backward pedaling at 80 RPM.

Synergies 2–5 of whole-speed walking showed similar muscle synergies across pedaling conditions, whereas synergy 1 did not exhibit similar muscle synergies in any pedaling condition.

### 3.5 Functions of representative muscle synergies for each condition

The functions of each representative muscle synergy for each condition are associated with their corresponding temporal patterns by classifying the primary muscle functions (*W* ≥ 0.5) (Rimini et al., 2017; Abd et al., 2022).

For whole-speed walking, synergy 1 was composed of the activity of Gmed during the early to mid-stance phase. Synergy 2 was characterized by the activity of the quadriceps group, Gmed, and Gmax during the early stance phase. Synergy 3 consisted of the activity of SOL and LG during the late stance phase. Synergy 4 involved the activity of TA during the swing phase, while synergy 5 included the activity of the hamstrings from the late swing to the early stance phase.

In pedaling, the cycle was divided into two phases: 0–50% for flexion and 50–100% for extension. Each synergy’s function was identified accordingly. In forward pedaling, synergy 1 showed increased activity during the early part of the flexion phase across all speeds. At 60 RPM, the hamstrings were the primary muscle, while at 30 RPM, both the hamstrings and Gmed contributed significantly. Conversely, synergy 1 at 80 RPM showed a significant contribution from RF, which differed from the other speeds. Synergy 2 consistently highlighted TA as the primary contributor across all speeds, with increased activity during the latter part of the flexion phase. Notably, at 30 RPM, RF also contributed significantly alongside TA. Synergies 3 at 60 RPM and 30 RPM, as well as synergy 4 at 80 RPM, primarily involved VM and VL, with activity increasing from the end of the flexion phase to the early part of the extension phase. Synergy 3 at 80 RPM exhibited isolated activity of VM. Synergies 4 at 60 RPM and 30 RPM, and synergy 5 at 80 RPM, primarily involved LG, functioning from the latter part of the extension phase to the early part of the flexion phase.

In backward pedaling, synergy 1 for each speed was mainly contributed by TA, remaining active from the flexion phase to the early part of the extension phase. Synergies 2 at 30 RPM and 80 RPM showed activity during the early part of the flexion phase, with the plantar flexor muscles contributing predominantly, although SOL’s contribution was minimal at 80 RPM. Synergy 2 at 60 RPM and synergy 3 at 80 RPM showed activity throughout the flexion phase, with significant contributions from the hamstrings. Synergies 3 at 60 RPM and 30 RPM, and synergy 4 at 80 RPM, primarily involved VM and VL, being active during the extension phase. Finally, synergy 4 at 60 RPM and 30 RPM, along with synergy 5 at 80 RPM, showed RF as the primary muscle, functioning from the latter part of the extension phase to the early part of the flexion phase.

## 4 Discussion

The novel finding of this study is that among the six pedaling conditions, comprising three rotational speeds (30 RPM, 60 RPM, and 80 RPM) and both forward and backward rotations, the identified muscle synergies included those similar to the muscle synergies observed during walking for each condition. The results of this study support the hypothesis that the majority of synergies are shared between walking and pedaling.

Different muscle synergies are associated with rotational direction and speed. In many subjects, the muscle synergy resembling that of walking, with predominant contributions from the plantar flexor muscles of the ankle, was recruited only during pedaling at 30 RPM, regardless of the direction of rotation. In contrast, the muscle synergy resembling that of walking, with predominant contributions from the hamstrings, was observed only during forward pedaling at 60 RPM and backward pedaling at 80 RPM. These findings reveal that specific walking-like muscle synergies are recruited only in certain directions and speeds.

### 4.1 Representative muscle synergies in whole-speed walking

This study identified five muscle synergies in walking. Among these, synergies 2–5 closely matched the characteristics of the four walking muscle synergies reported in previous studies (Neptune et al., 2009; Clark et al., 2010). Synergy 1, on the other hand, was uniquely identified in this study and primarily represented Gmed activity during the early to mid-stance phases. Because different sets of recorded muscles can result in different muscle synergy vectors (Turpin et al., 2021), the discrepancies between this study and previous studies may be due to the differences in the recorded muscle sets. Furthermore, given that the Gmed module is typically extracted during slow-to-moderate walking (Yokoyama et al., 2016), its inclusion in the synergies extracted from the EMG data encompassing a range of walking speeds in this study is considered valid.

### 4.2 Representative muscle synergies in forward pedaling

In forward pedaling, four representative muscle synergies were identified at 60 RPM and 30 RPM and five at 80 RPM, indicating variations in the number and composition of synergies depending on pedaling speed (Figure 3). In contrast to our study, previous research involving trained cyclists (Hug et al., 2011) reported that the number of synergies required to explain muscle activity during pedaling remained consistent at three, regardless of pedaling speed, and the synergy compositions were similar.

Cheung et al. (2020) reported that the number and composition of muscle synergies change plastically in response to developmental and training adaptations, suggesting that the differences in subjects between our study and previous studies might have influenced the number and composition of muscle synergies. Another study by Barroso et al. (2014) found that the EMG of all subjects while pedaling at four different speeds could be explained by four muscle synergies for untrained cyclists. Compared to the maximum speed of 70 RPM in the study by Barroso et al. (2014), our study included a higher pedaling speed of 80 RPM. This may explain the identification of a greater number of representative muscle synergies at higher speeds. Nevertheless, Barroso et al. (2014) reported distinct muscle synergy profiles at high and low speeds, which is consistent with our findings.

The composition of the muscle synergies identified at 30 and 60 RPM in our study closely resembled the four muscle synergies reported in previous pedaling studies (De Marchis et al., 2013; Barroso et al., 2014). At 80 RPM, muscle synergy involving the hamstrings, which are typically active during the early phase of the upstroke as observed at other speeds, was absent. Instead, the muscle synergy involving the rectus femoris (RF), which contributes to thigh lifting, appears during the early upstroke phase. Additionally, the muscle synergies of the knee extensors that were active during the downstroke phase were divided into two separate synergies. Previous research by De Marchis et al. (2013) indicated that inexperienced subjects predominantly adopt a pedaling strategy that adds propulsive force during the downstroke. Similar to previous research, our findings suggest that at a more demanding speed of 80 RPM, a propulsive force is generated by the activity of the knee extensors during the downstroke phase.

### 4.3 Representative muscle synergies in backward pedaling

The representative muscle synergies identified from backward pedaling also showed variations in the number and composition of muscle synergies depending on the pedaling speed (Figure 4). Additionally, four representative muscle synergies were identified at both 30 and 60 RPM. However, at 30 RPM, there was a larger individual variability in the composition of muscle synergies compared to other speeds, with many subject-specific muscle synergies observed. When examining the composition of the muscle synergy vectors at each backward pedaling speed, it appeared that the muscle synergy indicating the activity of the TA and RF observed in forward pedaling was split. At 80 RPM, five representative muscle synergies were identified, with the composition of muscle synergy vectors showing distinct synergies representing the activity of the TA, RF, plantar flexors, and hamstrings. A simulation study by Raasch and Zajac (1999) stated that the biarticular muscles of the thigh (rectus femoris and hamstrings) changed roles depending on the direction of movement, and smooth backward pedaling was achieved by controlling the pairs of TA/RF and hamstrings/plantar flexors separately, which were observed in forward pedaling. These results are consistent with the findings of the simulation study.

### 4.4 The similarity between walking muscle synergies and pedaling muscle synergies

In this study, 1–3 walking-like muscle synergies were present under all six pedaling conditions. The use of similar muscle synergies associated with different kinematic and kinetic patterns provides further evidence that the CNS generates movements through a flexible combination of muscle synergies (Tresch and Jarc, 2009). Similar to previous studies (Hug et al., 2010; De Marchis et al., 2013; Barroso et al., 2013, 2014), the results of this study suggest the existence of shared neural networks between walking and pedaling.

Furthermore, the results indicated that the composition of walking-like muscle synergies observed during pedaling depends on the direction and speed of pedaling. As shown in Figure 5, the muscle synergy associated with the activation of the quadriceps and hip extensors during whole-speed walking (synergy 2) was recruited during pedaling at 30 RPM, regardless of the direction of rotation. Meanwhile, the muscle synergy associated with the activation of the hamstrings (synergy 5) was recruited during forward pedaling at 60 RPM and backward pedaling at 80 RPM. Additionally, a muscle synergy similar to synergy 1, representing the activity of the Gmed and Gmax muscles during walking, was not observed under any pedaling condition. Walking synergy 1 is thought to contribute to pelvic stability during the stance phase. It is speculated that the minimal postural control required during pedaling may explain the lack of synergy 1 recruitment.

In contrast to the findings of this study, previous research on muscle synergies during upper-limb cycling reported a high degree of similarity and consistency in the number and structure of upper-limb synergies, regardless of power levels (Abd et al., 2022). One possible reason for the discrepancy between these studies is the difference in neural control between the upper and lower limbs. The upper limbs are involved in fine and diverse motor tasks, requiring precise control, whereas the lower limbs are specialized for posture control, weight-bearing, and cyclic movements, like walking and running. These differences in function may result in distinct neural control strategies, contributing to the different outcomes observed. Additionally, Abd et al. (2022) varied resistance load, while the present study examined the effect of rotational speed on muscle synergies under a constant resistance load. Since muscle synergies are recruited to optimize task performance (Tresch and Jarc, 2009; Bizzi and Cheung, 2013; Ting et al., 2015), differences in task conditions could explain the variation in results. Given the functional roles of the upper and lower limbs, further investigation into how rotational speed affects muscle synergies in the upper limbs is warranted.

### 4.5 Clinical application

Pedaling exercises, which involve muscle activity in the lower limbs similar to walking (Raasch and Zajac, 1999) and require minimal postural control, are a promising rehabilitation method for improving the walking ability of patients with stroke in the early stages of recovery when gait training is challenging (Barbosa et al., 2015). The results of this study suggest the importance of considering the direction and speed of rotation when adopting pedaling as a gait training method. For example, previous studies reported that changes in the recruitment of two synergies (TA and RF, TFL muscle synergy; hamstrings and plantar flexors, Gmax muscle synergy) characterizing the upstroke phase of forward pedaling on the paretic side of patients with stroke are positively correlated with indicators of gait asymmetry (Ambrosini et al., 2016). The results of this study suggest that forward pedaling is effective for selectively recruiting the TA and RF muscle synergy (synergy 4), regardless of speed (Figure 5). Alternatively, forward pedaling at 60 RPM and backward pedaling at 80 RPM may be more suitable for selectively recruiting the hamstrings and MG muscle synergy (synergy 5). Therefore, by considering the direction and speed of pedaling according to the patient’s impairment, it may be possible to enhance its effect on improving walking function.

### 4.6 Limitations

The EMG activity of each muscle was normalized to the average peak value across six cycles for each condition. This method is similar to those used in previous studies involving muscle synergies (Hug et al., 2011; Barroso et al., 2014; Ambrosini et al., 2016), but since the muscle activity levels are provided only as relative information to the peak values, it is not possible to directly quantify the contribution of power output from each muscle synergy. However, a standardized normalization method that accurately quantifies the contribution of the output from each muscle synergy has not yet been established (Ambrosini et al., 2016). Moreover, because the number and composition of muscle synergies during pedaling in patients with stroke differ from those in healthy individuals (Ambrosini et al., 2016), it is necessary for future research to elucidate how walking-like muscle synergies observed during pedaling are affected by the speed and direction of rotation in these patients.

## 5 Conclusion

The present study indicated common muscle synergies between walking and pedaling. However, the composition of similar muscle synergies varied with pedaling speed and direction. Our results suggest that it is crucial to consider muscle synergy when performing pedaling exercises for gait rehabilitation.

## Data Availability

The raw data supporting the conclusions of this article will be made available by the authors, without undue reservation.
